# Unveiling GFAP Astrocytopathy: Insights from Case Studies and a Comprehensive Review of the Literature

**DOI:** 10.3390/antib13040079

**Published:** 2024-09-25

**Authors:** Panagiotis Gklinos, Fotios Athanasopoulos, Vagia Giatrakou, Nikolaos-Achilleas Arkoudis, Dorothea Pournara, Eirini Giagkou, Argyro Tountopoulou, Sofia Vassilopoulou, Dimos-Dimitrios Mitsikostas

**Affiliations:** 1 First Neurology Department, Eginition University Hospital, National and Kapodistrian University of Athens, 11528 Athens, Greecedmitsikostas@med.uoa.gr (D.-D.M.); 2 Research Unit of Radiology and Medical Imaging, National and Kapodistrian University of Athens, 11528 Athens, Greece; 3 The Second Department of Radiology, General University Hospital “Attikon”, National and Kapodistrian University of Athens, 12462 Chaidari, Greece

**Keywords:** glial fibrillary acidic protein astrocytopathy, antibody, autoimmune diseases, neuroinflammation, meningoencephalitis, autoimmune encephalitis, paraneoplastic encephalitis

## Abstract

Background: Autoimmune glial fibrillary acidic protein (GFAP) astrocytopathy, which was first identified in 2016, is an immune-mediated inflammatory disorder of the nervous system characterized by antibodies targeting GFAP. The exact pathogenic mechanisms, as well as the role of anti-GFAP antibodies, remain unclear; however, it seems that neuroinflammation is mediated by specific CD8+ T-cells and that neoplasms or viral infections can act as the initial trigger. Although the clinical spectrum of the disease is broad and heterogenous, GFAP astrocytopathy most commonly presents as meningoencephalitis with or without myelitis. Other symptoms include headache, visual disturbances, extrapyramidal or brainstem syndromes, and psychiatric manifestations including psychosis. The disease has a characteristically favorable response to steroid treatment while relapses occur in approximately 20–30% of the patients. Methods: We present two cases of GFAP astrocytopathy admitted to our hospital: a 43-year-old male with persistent headache and a 59-year-old female with acute dysarthria and swallowing difficulties followed by cognitive and behavioral symptoms. Results: Additionally, we conduct a comprehensive review of the literature to elucidate the role of anti-GFAP antibodies in disease pathogenesis and examine imaging characteristics, clinical manifestations, and treatment options for this recently described neuroimmunological condition. Conclusions: This review presents two unusual cases of GFAP-astrocytopathy and provides evidence for the pathogenesis, clinical presentation, imaging characteristics and treatment options of the disease.

## 1. Introduction

Glial fibrillary acidic protein (GFAP) astrocytopathy, which was first described in 2016, is an immune-mediated, inflammatory disease of the central nervous system (CNS) characterized by the presence of IgG autoantibodies against the intermediate filament protein GFAP (especially GFAPα isoform) in the cerebrospinal fluid (CSF) [1]. Both tissue-based and cell-based immunofluorescence assays are used for the detection of GFAP-IgG [2]. A deeper understanding of the etiology and pathogenetic mechanisms of GFAP astrocytopathy is still lacking; however, it often coexists with a malignancy (most commonly ovarian teratoma), anti-neuronal and anti-glial autoantibodies, while a link between viral infection and GFAP astrocytopathy has also been reported in several case series [1,2,3,4,5,6,7]. Viral infections could activate the immune system through molecular mimicry leading to anti-GFAP antibody production, activation of GFAP-specific CD8+ T-cells, blood–brain barrier disruption, and cellular cytotoxicity leading to target cell apoptosis. Similarly, tumor cells might express antigens that resemble GFAP, which cross-react with astrocytic GFAP, and induce T-cell mediated autoimmunity, which finally results in neuronal damage. The co-existence of anti-glial or anti-neuronal antibodies constitutes a negative prognostic factor [3]. In particular, accompanying anti-NMDA-r antibodies, anti-AQP4 antibodies, or concomitant malignancy are associated with poorer response to steroids, higher relapse rates, and poorer long-term prognosis [3].

The onset of the disease is acute or subacute, and it is typically diagnosed in individuals over 40 years old. Clinical manifestations include symptoms of meningoencephalitis or meningoencephalomyelitis such as fever, headache, neck stiffness, and epileptic seizures, while other symptoms such as tremors, sensory disturbances, autonomic dysfunction, and psychiatric manifestations have been reported [1,2,3,4,5,6]. The imaging hallmark of GFAP astrocytopathy is brain linear perivascular radial gadolinium enhancement on magnetic resonance imaging (MRI) [2,8,9]. Most cases of GFAP astrocytopathy respond favorably to high-dose corticosteroids; however, treatment-resistant or relapsing cases that require additional treatment with intravenous immunoglobulin (IVIG) and plasma exchange or steroid-sparing agents such as rituximab, mycophenolate mofetil, azathioprine, and cyclophosphamide have been reported [2,3,4,5,10].

Although a number of case series have emerged over the past few years, various aspects of the disease, such as the pathogenetic mechanisms, treatment recommendations, and prognosis, remain largely obscure. Herein, we describe two cases of GFAP astrocytopathy and provide a comprehensive review of the literature, aiming to describe the pathogenesis, clinical spectrum, and imaging characteristics of the disease, as well as explore the acute and preventive therapeutic options.

## 2. Case Reports

### 2.1. Case 1

**Figure 1:**
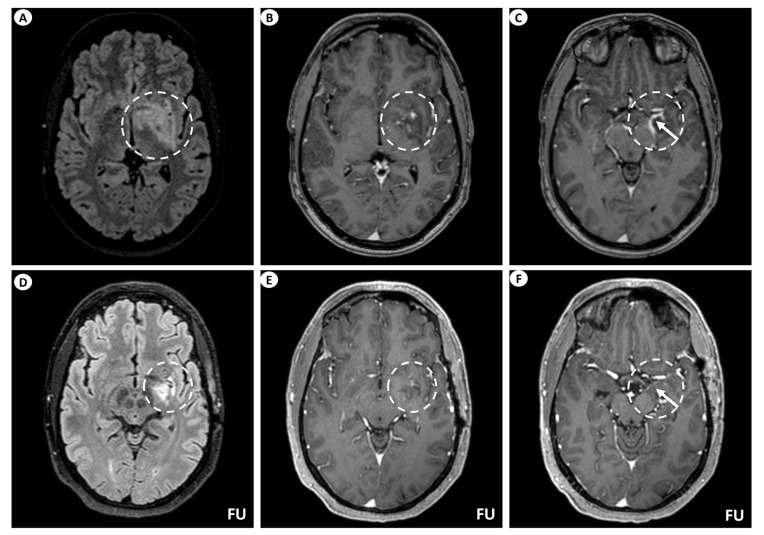
Brain MRI of patient 1. (**A**) Axial T2-FLAIR (fluid-attenuated inversion recovery) images display signal hyperintensity centered within the basal ganglia (putamen, globus pallidus) and internal capsule on the left (dashed circle). (**B**) Axial T1-weighted images following intravenous gadolinium injection demonstrate punctate as well as linear contrast enhancement extending along the perivascular spaces (dashed circle), and (**C**) leptomeningeal contrast enhancement along the surface of the uncus (as indicated by the arrow within the dashed circle). Follow-up (FU) MRI examination in the same patient displays a noteworthy decrease in the extent of the signal hyperintensity within the left basal ganglia on axial T2-FLAIR images (dashed circle) (**D**). Similarly, axial T1-weighted images following intravenous gadolinium injection demonstrate partial subsidence of the perivascular contrast enhancement (dashed circle) (**E**), and complete resolution of the leptomeningeal enhancement (arrow within the dashed circle) (**F**).

A 44-year-old man presented with persistent, left-sided, daily headache episodes of tightening quality and medium severity (visual analog scale score 6/10) over the past 15 days. No prior history of headaches was reported, and his past medical history was insignificant except for smoking (40 pack-years). Neurological examination was completely normal. Magnetic resonance imaging (MRI) of the brain revealed T2/fluid-attenuated inversion recovery (FLAIR) signal hyperintensity within the basal ganglia (putamen, globus pallidus), internal capsule, medial temporal lobe, hypothalamus, and midbrain on the left. Linear perivascular radial gadolinium enhancement was also observed, extending along the perivascular spaces in these areas, as well as leptomeningeal contrast enhancement along the surface of the uncus (Figure 1). The patient did not have a lumbar puncture but instead underwent a brain biopsy, which was inconclusive, and received a short course of oral steroids following the biopsy. He was subsequently lost to follow-up but was eventually readmitted for further investigations. At that time, he underwent a lumbar puncture, which showed no pleocytosis. Cerebrospinal fluid (CSF) protein, glucose levels, and IgG index were within normal ranges, while oligoclonal bands were negative. However, it is important to note that the lumbar puncture was performed 6 months after symptom onset and following steroid treatment. A second brain MRI showed significant improvement, with remission of the hyperintensities and of the contrast enhancement (Figure 1), while an MRI of the spinal cord did not reveal any signs of myelopathy or other abnormal findings. CSF and serum testing for GFAP antibodies returned positive, while antibody testing for autoimmune and paraneoplastic encephalitis, including surface and intracellular antibodies, was negative. The patient underwent computerized tomography (CT) of the chest and abdomen, which was normal, as well as a positron-emission tomography (PET) scan, which was negative for malignancy. Subsequently, he received a five-day course of intravenous (IV) steroids without tapering and has remained asymptomatic since then.

### 2.2. Case 2

A 59-year-old female patient presented with a subacute onset of speech and swallowing difficulties, followed shortly by behavioral disturbances that evolved over the past two weeks. Neurological examination revealed psychomotor agitation and aggression as well as dysarthria and dysphagia. Her medical history was significant for arterial hypertension and chronic alcoholism. Magnetic resonance imaging (MRI) of the brain showed T2/(FLAIR) non-specific hyperintensities in the subcortical white matter and periventricular regions without any gadolinium enhancement. The electroencephalogram (EEG) showed slow theta and delta waves. CSF analysis showed pleocytosis (62 white blood cells), increased protein levels (84 mg/dL), and a normal CSF/serum glucose ratio. Additionally, the IgG index and oligoclonal bands were positive. Differential diagnosis included autoimmune or paraneoplastic encephalitis and the patient was treated with a five-day course of intravenous methyprednisolone, without improvement. A repeated lumbar puncture revealed slight improvement (40 WBC, 71 mg/dL protein). Subsequently, CSF and serum testing for GFAP antibodies returned positive, while the panel for other autoimmune and paraneoplastic encephalitis was negative. Given the lack of response to steroid treatment and the severity of the syndrome, the patient underwent five cycles of plasma exchange over 10 days, which resulted in immediate clinical improvement in speech and behavioral disturbances, while swallowing became possible. Laboratory results also showed improvement (20 cells in CSF analysis), as did the EEG findings (only a few theta waves without the presence of delta waves). A CT scan of the chest and abdomen showed a right adrenal mass with internal calcifications and enrichment on the positron-emission tomography (PET) scan. Unfortunately, a biopsy was not performed because the patient died due to septic shock from a urinary infection.

The data and images included in this report were obtained through standard clinical procedures. The patients underwent a full neurological assessment, which included clinical examinations, MRI scans, and laboratory investigations. Consent for the use of clinical data and images was obtained from both patients. The MRI images were acquired using 3D T2, 3D FLAIR, 3D T1 pre- and post-gadolinium, DWI and SWI sequences for brain MRI, and axial/sagittal T2, sagittal/coronal STIR, and axial/sagittal T1 pre- and post-gadolinium sequences for spinal MRI, ensuring detailed visualization of the findings. All procedures followed institutional ethical guidelines.

## 3. History

In 2016, cerebrospinal fluid (CSF) of four patients with autoimmune encephalitis associated with ovarian teratoma produced a novel, filament-like, IgG staining pattern of adult mouse cerebrum by indirect immunofluorescence assay (IFA) as part of he Mayo Clinic Neuroimmunology Laboratory’s extramural diagnostic serological practice [1]. This pattern was mostly observed within specific brain areas including pial, subpial, subependymal, perivascular, and periventricular regions. Concurrently, CSF analysis of a second group of patients with chronic, relapsing, steroid-responsive meningoencephalitis, which was initially named chronic microglial encephalomyelitis due to the abundance of microglia within chronic inflammatory brain lesions, revealed the same immunohistochemical characteristics as the autoimmune encephalitis group [11]. The above-mentioned laboratory and clinical observations led to the description of the characteristic GFAP staining pattern and the associated disease was named GFAP astrocytopathy [2].

The filament-like staining pattern observed in the subventricular (lateral) zone suggested that an intermediate filament protein might be the antigen of interest [1]. Subsequently, Western blot analysis revealed a 50 kDa protein band, common among the patients studied, which led to the identification of GFAP. This band was further analyzed by mass spectrometry. To replicate the IFA pattern, patient IgG was extracted from the 50 kDa protein band and was applied to mouse tissue. A GFAP-transfected HEK293 cell-based assay was used to validate the antigen specificity, as well as to investigate whether patient IgG specifically targeted specific isoforms such as GFAP-δ/ε, GFAP-α, or shared isoform epitopes. At first, an immature GFAP isoform, such as δ/ε or k, was expected to be the one that was most commonly targeted; however, all patients exhibited GFAP-IgG that reacted with the mature (a) GFAP isoform. Antibodies targeting one or both of the GFAP d/e or k isoforms were identified in 80% of the patients, though these were never found alone. The specific GFAP isoform neither affected the clinical phenotype nor was associated with a history of cancer or infection.

## 4. Epidemiology and Demographics

Due to the recent identification of GFAP astrocytopathy in 2016, insight into its epidemiology and demographics remains very limited. However, it is considered a rare disease, despite being underdiagnosed. Although the incidence and prevalence of the condition remain unspecified, a study on the population of Olmsted County, Minnesota reported an incidence of 0.03 per 100,000 person-years and a prevalence of 0.6 per 100,000 people [12]. GFAP astrocytopathy most commonly occurs in adults with only approximately 10% of the cases concerning children, who present with similar clinical manifestations to those of adults [13]. The median age at symptom onset is 42–54 years [1,2,3,4]. Sexes are affected almost equally, but according to some studies, there might be a slight female predominance [2,14]. To date, there are no data supporting any racial predisposition; however, it must be noted that the patients included in the available studies are predominantly Caucasian and Asian. Similar to other rare neurological disorders, the underrepresentation of specific ethnicities in the existing case series may affect our understanding of the epidemiological and demographical characteristics of the disease.

## 5. GFAP-Antibodies Detection Methods

GFAP is a cytoplasmic, intermediate, filament protein of the astrocytes with more than eight isoforms. All of them have identical N-terminal head and coiled-coil rod domains, but divergent C-terminal tails [1].

GFAP-IgG detection includes tissue-based assays (TBA), cell-based assays (CBA), and Western blot. Most patients with GFAP astrocytopathy clinically present with meningoencephalitis and/or myelitis and have GFAP-IgG either in the CSF and serum or only in the CSF. The presence of GFAP-IgG in the CSF only suggests a CNS-restricted syndrome, which is typical for GFAP astrocytopathy. On the other hand, detection of GFAP-IgG in both CSF and serum suggests a broader immune response and could possibly correlate with a more severe clinical presentation. CSF analysis is more sensitive for the detection of GFAP antibodies than serum.

Experts suggest that a two-step process that includes the detection of GFAP-IgG through tissue-based immunohistochemistry, followed by the detection of GFAP-IgG through transfected cell-based immunofluorescence assays to increase specificity, is the gold standard for the diagnosis of GFAP astrocytopathy [2]. Cryosections of rodent brains are used as substrates for tissue-based immunofluorescence assays. CSF from GFAP astrocytopathy patients is immunoreactive against specific brain areas including pial, subpial, and periventricular regions, while cerebellum, brainstem, and cerebral white matter are less commonly involved. TBA testing alone can lead to non-specific results (false positive), while CBA testing for all eight isoforms is considered challenging and can lead to false negative results since, rarely, some patients can be GFAPα-IgG negative but positive for other isoforms, such as GFAPε-IgG [13,14].

## 6. Pathophysiology

**Figure 2:**
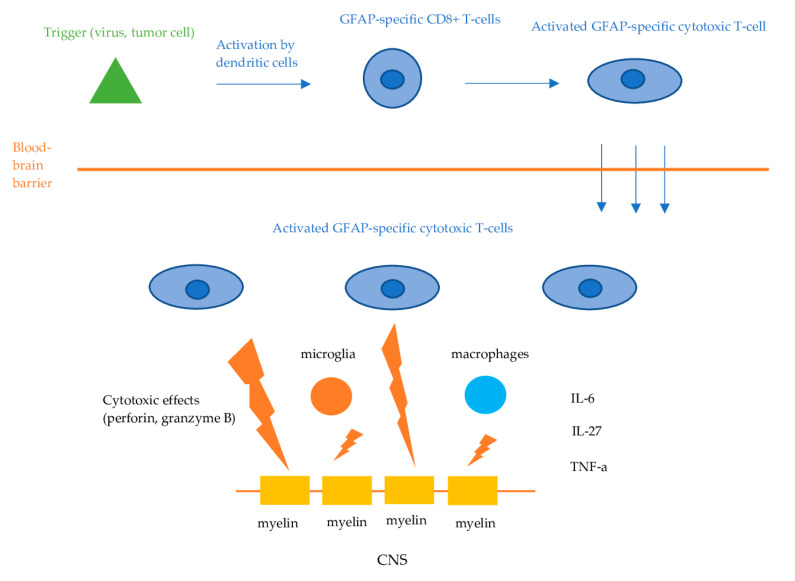
Proposed pathophysiologic mechanisms of GFAP astrocytopathy. Dendritic cells phagocytose tumor- or virus-associated antigens and present them to lymphocytes in the regional lymph nodes. This process activates antigen-specific CD8+ cytotoxic T-cells, which then infiltrate the central nervous system. Upon encountering these antigens displayed on the cell surface by MHC-I molecules, the T-cells bind and exert their cytotoxic effects via the release of perforin and granzyme B, leading to the apoptosis of the target neuronal cells.

Information on the exact causative mechanisms of GFAP astrocytopathy is still lacking. GFAP is an intracellular, cytoplasmic filament protein of the astrocytes, inaccessible by circulating IgG when astrocytes remain intact. Therefore, anti-GFAP antibodies are not considered to be directly pathogenic. Like other autoimmune encephalitis with antibodies against intracellular antigens, the effect of GFAP antibodies is presumed to be mediated via cellular immunity, namely, GFAP-specific CD8+ T-cells [1,2,4,5]. The initial trigger (viral or tumor-associated protein) is phagocytosed and presented by dendritic cells to lymphocytes in regional lymph nodes in the periphery. This leads to the activation of the GFAP-specific CD8+ T-cells, which then invade the CNS and exert their cytotoxic effects, resulting in the apoptosis of the target cells (Figure 2). However, in some cases, the initial trigger remains unknown. Mouse models demonstrated that GFAP-reactive CD8+ T cells resulted in distinct characteristics of inflammatory CNS autoimmunity, depending on the initial event that triggered the cellular response [15]. Besides CD8+ T cells, components of the innate immune system, such as microglia and macrophages, as well as regulators of inflammation and immune responses including a variety of cytokines and chemokines, have been suggested to play an important role in the pathophysiological cascades of GFAP astrocytopathy [2]. Tumor necrosis factor alpha (TNF-a), interleukin (IL)-27, IL-6, and chemokine [C-C motif] ligand 20 CSF levels have been found to be elevated in patients with GFAP astrocytopathy, while significant association between those cytokines, chemokines, and CSF levels of biological markers, including GFAP, neurofilament light chain (NfL), and S100 calcium-binding protein B, has also been observed [16].

In regard to etiology, several theories have attempted to explain GFAP astrocytopathy. Importantly, GFAP ectopic expression by neoplasms has been thought to trigger an immune response resulting in a paraneoplastic autoimmune reaction directed against the self-antigen (GFAP) [2]. Indeed, approximately 34% of patients with GFAP astrocytopathy were diagnosed with neoplasms either at the time of presentation or in the years that followed. Ovarian teratoma was by far the most common (70%) and had the strongest association, followed by adenocarcinoma of endometrium, esophagus and kidney (15%), glioma (10%), squamous cell carcinoma and multiple myeloma [2]. In another study, only 13% of the patients were diagnosed with neoplasms, including ovarian teratoma (33%), breast cancer (33%) and thymoma (33%) [4]. Other neoplasms that have been reported in patients with GFAP astrocytopathy include head and neck squamous cell carcinoma, pleomorphic parotid adenoma, small-cell carcinoma, and carcinoid [2,3,4,5].

Furthermore, administration of specific treatments affecting T-cell regulation such as immune checkpoint inhibitors (ipilinumav, nivolumab) for the treatment of neoplasms or the anti-CD25 monoclonal antibody daclizumab, which was, for a short period of time, used for the treatment of multiple sclerosis (MS), has been associated with cases of GFAP astrocytopathy [2,12,17]. These observations further suggest that the effect of GFAP antibodies is probably mediated through GFAP-specific CD8+ T-cells.

The increased occurrence of infections prior to the onset of neurological symptoms in patients with GFAP astrocytopathy has fueled discussions around a parainfectious mechanism. Iorio et al. (2018) reported that 27% of the patients presented with prodromal symptoms including fever of unknown origin, dengue fever, interstitial pneumonia, and diarrhea, and were initially diagnosed with an infectious disease [4]. Moreover, Flanagan et al. (2017) reported prodromal, flu-like symptoms in 29% of the patients including upper or lower respiratory tract infections, urinary tract infections, and prostate infection [2]. In addition, herpes simplex virus (HSV) infection has been reported by Long et al. (2015) [5]. A meta-analysis by Hagbohm et al. (2024) reported a viral prodromal infection in 45% of the patients [18]. Most commonly, this was an upper respiratory tract or a gastrointestinal infection, though, the human immuno-deficiency virus and bacterial infections had been rarely reported. Although data from the above-mentioned studies indicate a possible role of infectious agents in the pathogenesis of GFAP astrocytopathy, the exact mechanism remains largely unknown.

Finally, coexisting anti-neuronal and anti-glial antibodies, including NMDA-R-IgG and AQP4-IgG, have been observed in patients with GFAP astrocytopathy. More specifically, Long et al. (2018) reported that 33% of patients were positive either for NMDA-R-IgG or AQP4-IgG [5]. Noticeably, Flanagan et al. (2017) reported that teratoma was diagnosed in 71% of patients with the coexistence of NMDA-R–IgG and AQP4-IgG and 53% of patients with the coexistence of NMDA-R–IgG alone. Teratoma was also diagnosed in 66% of patients with the coexistence of AQP4-IgG alone [2]. Other coexisting antibodies that have been reported include myelin oligodendrocyte glycoprotein (MOG) and glutamic acid decarboxylase 65 (GAD65). AQP4-IgG or MOG-IgG coexistence does not seem to influence the clinical phenotype of the disease, which is very similar to patients with GFAP-IgG alone. However, rare cases of neuromyelitis optica spectrum disorder (NMSOD) have been reported [2,10]. On the contrary, the coexistence of NMDA-R-IgG might affect disease phenotype with a clinical course similar to NMDA-R encephalitis with limbic encephalitis and prominent psychiatric manifestations [2].

## 7. Clinical Spectrum

Most patients with GFAP astrocytopathy present with acute or subacute meningoencephalitis/meningoencephalomyelitis. A large meta-analysis, which pooled data from 492 patients, reported that 32% developed meningoencephalomyelitis, 24% had meningoencephalitis, 12% presented with encephalitis, 12% with encephalomyelitis, 5% with myelitis, and 4% with meningitis [18].

Initial symptoms include fever, headache, and nausea mimicking CNS infection, commonly followed by symptoms of meningoencephalitis including encephalopathy, decreased level of consciousness, and meningeal signs. Rarely, seizures can also be a presenting manifestation.

Although meningoencephalomyelitis is the most common presenting clinical syndrome, GFAP astrocytopathy is characterized by broad and heterogenous symptomatology. Other less commonly encountered clinical findings include movement disturbances encompassing tremor, dyskinesia, myoclonus, and chorea, cognitive impairment including predominantly executive dysfunction but also memory deficits, and psychiatric symptoms including depression, anxiety, and psychosis [2,5,19]. Involvement of the cerebellum or the brainstem is not rare with about 22–57% of the patients presenting with ataxia, 16–37% presenting with eye movement abnormalities, and 5% having swallowing dysfunction [2,3,19,20]. Involvement of the brainstem could also result in area postrema syndrome in about 11% of the patients [18,21]. Deng et al. (2022) reported that area postrema syndrome in GFAP astrocytopathy frequently appeared in the early stages of the disease, similar to its occurrence in AQP4 + NMOSD [21]. Contrary to AQP4 + NMOSD, hiccups were the most common symptom instead of nausea or vomiting. Another difference is that in GFAP astrocytopathy, area postrema syndrome never appeared as an isolated syndrome, whilst it accounted for 33% of episodes in NMOSD.

Visual system involvement is heterogenous and is encountered in approximately 28% of the patients [18]. It ranges from asymptomatic bilateral optic disk edema to severe, bilateral, treatment-resistant vision loss [22]. According to a systematic review of the literature conducted by Greco et al. (2023), symptomatic or asymptomatic visual system involvement was reported in 25% of the patients [22]. Bilateral optic disk edema was very common and was reported in 50% of the patients (61% of them were asymptomatic). Symptoms were reported in 17% of the patients. However, instead of experiencing the painful vision loss associated with optic neuritis, patients reported blurred vision or transient visual obscurations due to optic disk edema, mimicking papilledema. According to the same systematic review, optic neuritis was reported in only 6% of the patients and often supported by inconsistent clinical or paraclinical data. A retrospective observational study by Chen et al. (2018) reported that 25% of GFAP astrocytopathy patients had optic disk edema, which was bilateral in all cases [23]. However, CSF opening pressure was typically normal or slightly elevated, hence, making it unlikely to be the primary cause of the optic disk edema. Visual acuity was unaffected, while visual field tests showed only minor arcuate defects. Ocular coherence tomography (OCT) indicated thickening of the retinal nerve fiber layer, while fluorescein-based retinal angiography appeared normal [23]. Given that opening pressure is usually normal, the precise mechanism of optic disk edema remains elusive. A theory that has been suggested involves the targeting of GFAP in the retina, particularly in the end feet of Müller cells and astrocytes, which can lead to the breakdown of the retina–blood–brain barrier, resulting in optic disk edema [24]. In conclusion, GFAP astrocytopathy can cause optic disk edema, which is very often bilateral and can result in blurred vision, though in most cases is asymptomatic, while optic neuritis is very rare. Patients with meningoencephalomyelitis and optic disk edema and/or visual disturbances should be tested for GFAP antibodies, particularly when AQP4 and MOG antibodies are negative.

Spinal cord involvement in GFAP astrocytopathy is commonly observed as part of meningoencephalomyelitis or encephalomyelitis syndrome (42%), rather than as isolated myelitis (5%) [18]. Moreover, symptoms are usually distinct from typical cases of myelitis. Kunchok et al. (2019) reported that patients with GFAP astrocytopathy more frequently experience myelopathic symptoms including sensory and mild motor symptoms, rather than severe paresis/plegia from myelitis, in contrast to patients with AQP4 NMOSD [19].

Another commonly reported symptom is autonomic dysfunction. Approximately 38% of patients experience dysautonomia, including bowel or urinary disturbances, while erectile dysfunction and blood pressure fluctuations were reported more rarely [18,19,20,25]. Lan et al. (2023) suggested that in cases of acute onset encephalitis or meningoencephalitis presenting with headache, fever, meningeal signs, decreased consciousness, tremor, and urinary/bowel disturbances, there is a high risk of detecting anti-GFAP antibodies [25].

Recently, there has been an increasing number of studies reporting patients with peripheral neuropathy. The rate of peripheral neuropathy in patients with GFAP astrocytopathy ranges from 24% to 31% [18,21,25]. Peripheral neuropathy in GFAP astrocytopathy is mostly axonal, predominantly affecting the lower motor extremities [21]. However, some studies have also reported peripheral neuropathy with decreased motor and sensory nerve conduction velocities and amplitudes [25]. To our knowledge, there has been no patient with isolated peripheral neuropathy without central nervous system involvement so far.

Lastly, it should be noted that approximately 12% of patients will experience significant neurological deterioration, leading to respiratory failure, coma, and intubation [5,18,20]. GFAP astrocytopathy associated clinical phenotypes and symptoms are summarized in Table 1. 

## 8. Imaging Characteristics

In terms of the role of imaging studies, magnetic resonance imaging (MRI) examinations may frequently demonstrate abnormal findings, raising suspicion of GFAP astrocytopathy and assisting in an appropriate diagnosis.

The most commonly encountered imaging manifestations include T2 signal abnormalities in the periventricular white matter. This finding’s presence has been documented in as low as 11.8% [9] and up to 75% [1] of patients. Nonetheless, besides the periventricular white matter, T2 signal abnormalities may also be recognized, affecting various different regions such as the basal ganglia, the thalami, the cortex, the brainstem, the cerebellum, and even the hippocampus. Specifically, in a 2021 study by Xiao et al. [9], basal ganglia and thalamic lesions were displayed in up to 41.2% of the patients, cortical lesions were displayed in 29.4% of the patients, while lesions were also depicted in the brainstem (23.5%), the cerebellum (5.9%), and the hippocampus (5.9%). Similarly, in a recent relevant study by Ke et al. (2024) [26], the evaluation of brain MRI examinations in 33 patients with GFAP astrocytopathy demonstrated T2/FLAIR abnormal hyperintensities in the vast majority of the cases studied (90.9%), most usually with a juxtacortical (54.5%) or a periventricular (48.5%) white matter localization, followed by lesions in the basal ganglia (45.5%), the brainstem (33.3%), the thalami (27.3%), and the cerebellum (6.1%). However, T2 periventricular white matter hyperintensities are a largely unspecific imaging finding.

On this note, a remarkable imaging finding considered a hallmark of GFAP astrocytopathy, which can be detected after intravenous gadolinium contrast agent injection, is a linear perivascular pattern of enhancement, which is usually located in the periventricular white matter in GFAP-enriched regions and extends outwards in a radial pattern [27,28]. This significant finding has been documented in as low as 23.5% [9] to up to 53% [2] of patients. Other manifestations of cerebral enhancement patterns may include leptomeningeal enhancement, encountered in as low as 33% [1] to up to 45.5% of patients [18], periependymal enhancement [28], as well as punctate or serpentine enhancement [4].

Moreover, GFAP astrocytopathy has also been shown to produce optic nerve abnormalities [9,22] and papillitis [28].

Likewise, GFAP astrocytopathy may also affect the spinal cord, producing signal abnormalities and abnormal enhancement [28]. Specifically, patients with clinical suspicion of myelitis may demonstrate extensive long-segment myelitis, illustrated as T2 signal hyperintensity with a caudocranial extension of more than three vertebral bodies with or without concomitant contrast enhancement with a linear, punctate, or patchy pattern [27]. To further analyze, in a study by Fang et al., a longitudinally extensive increase in the T2 signal was detected in up to 71% of patients with myelopathy, although up to 29% had a normal spine MRI study [1]. In the study by Xiao et al. on 15 patients undergoing spine MRI, 53.3% displayed the typical finding of longitudinal myelitic lesions, 26.7% had short myelitic lesions, and up to 20% had a normal spine MRI study. Central canal or leptomeningeal contrast enhancement was seen in 66.7% and 13.3% of patients, respectively [9]. In the study by Ke et al. (2024), an abnormal T2 hyperintense signal in the spinal cord was displayed in 19/27 cases undergoing spine MRI (70.4%), more commonly affecting long segments (55.6%) and the thoracic spinal cord (25.9%). Moreover, in the cases undergoing intravenous gadolinium injection, abnormal spinal cord enhancement was seen in 59.1%, most frequently exhibiting a leptomeningeal pattern of enhancement (54.5%), followed by enhancement in the central spinal canal (36.4%) [26].

Generally, following appropriate treatment, patients typically exhibit subsidence in imaging findings regarding brain and spine abnormal signal hyperintensity and abnormal enhancement [9,26]. Nonetheless, some lesions may not demonstrate improvement or may even worsen [9], while lesions can also relapse [14].

**Figure 3:**
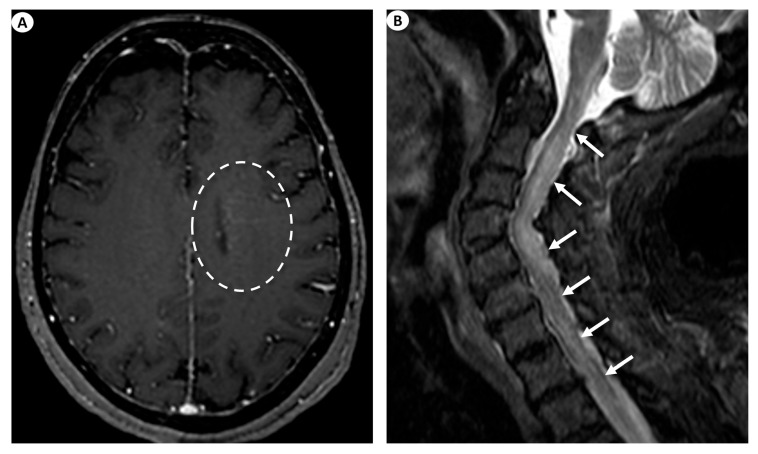
(**A**) Axial T1-weighted contrast-enhanced images following intravenous gadolinium injection demonstrate subtle periventricular linear contrast enhancement on the left cerebral hemisphere extending outwards in a radial pattern in a patient with GFAP astrocytopathy (dashed oval). (**B**) Sagittal fat-saturated T2-weighted images display abnormal signal hyperintensity extending craniocaudally from the cervicomedullary junction throughout the whole cervical spinal cord to the thoracic spinal cord (more than three vertebral levels affected), findings indicative of longitudinally extensive myelitis (arrows) in a female patient with GFAP astrocytopathy.

Examples of the aforementioned descriptions are illustrated in Figure 1 and Figure 3.

Coexisting neural autoantibodies detected in GFAP astrocytopathy cases may further complicate the diagnosis. Paraneoplastic causes could potentially be responsible [2,14], and ovarian teratomas answer as the most commonly coexisting neoplasm [13]. Thus, it is apparent that the role of imaging may extend to encompass the detection or exclusion of concurrent conditions during the workup process for GFAP astrocytopathy.

In terms of differential diagnosis, imaging findings of GFAP astrocytopathy may demonstrate substantial similarities with various diverse conditions. Perivascular enhancement in a radial pattern extending to the ventricles has been described in cases of neurosarcoidosis, mostly due to the inflammation surrounding deep medullary veins [29,30]. Isolated sarcoidosis CNS involvement is rare, and the disease usually affects other systems, most commonly the chest and lymph nodes [31]. Furthermore, on imaging, amongst various manifestations, neurosarcoidosis will commonly involve the dura matter and the meninges, producing typical dural and leptomeningeal enhancement, respectively, while usually favorably involving the basal cisterns [32,33]. In addition, this pattern can also be encountered with CNS vasculitis, intravascular (or angiocentric) lymphoma, and CLIPPERS (chronic lymphocytic inflammation with pontine perivascular enhancement responsive to steroids), all of which should be considered in the differential diagnosis of perivascular enhancement [27]. Imaging clues pointing towards the diagnosis of angiocentric lymphoma will include abnormal T2/FLAIR signal abnormalities with a dynamically alternating pattern, ischemic lesions and microhemmorrhages, parenchymal areas with expanding enhancement, and leptomeningeal and ependymal enhancement [34]. Furthermore, in cases of CNS vasculitis, in addition to perivascular enhancement, imaging may also depict ischemic lesions, microhemorrhages, and the actual involvement of vessel walls, seen as alternating stenosis and dilatations, all of which are findings uncharacteristic of GFAP astrocytopathy [35]. Finally, although CLIPPERS has been traditionally known to be encountered in the posterior fossa (pontine perivascular enhancement), a variation affecting the supratentorial brain (SLIPPERS) has also been observed [36,37]. Due to their demonstration of perivascular enhancement, these conditions should also be included in the differential diagnosis and may be difficult to exclude given that cortisone responsiveness is not unique to this disorder.

To conclude, the imaging findings of GFAP astrocytopathy are not pathognomonic. Nonetheless, based on imaging and in the appropriate clinical scenario, the diagnosis should be entertained if the typical perivascular radial enhancement pattern is encountered.

## 9. Treatment and Prognosis

To date, randomized controlled clinical trials regarding the proper management of the acute symptoms of GFAP astrocytopathy are lacking. However, similar to other autoimmune encephalitis or encephalomyelitis, earlier diagnosis and treatment initiation lead to better outcomes [16]. The responsiveness of GFAP astrocytopathy to corticosteroid administration is a consistent observation and constitutes a hallmark of the disease [2,12,13,16,25]. A recent meta-analysis, which included pooled data from 342 patients, revealed that 302 patients (88%) responded very well to high-dose intravenous corticosteroids, showing partial or complete remission with significant improvement in modified Rankin scale (mRS) scores, even in those who required intubation due to brainstem involvement [18]. These results are in line with those of Xiao et al., who reported an 82.8% improvement following intravenous methylprednisolone (IVMP) administration [9]. This favorable response to high-dose intravenous corticosteroids establishes them as the most frequently chosen treatment during the acute phase. It is hypothesized that dendritic cells, which present GFAP peptides to lymphocytes, are sensitive to corticosteroids, a fact that partly explains this responsiveness [4]. Additionally, Liao et al. (2022) suggested that the rapid remission of meningeal enhancement could serve as an early sign of response to treatment [38].

In more severe cases or in patients not responding to IVMP, alternative first-line immunotherapies such as intravenous immunoglobulin (IVIG) and/or plasma exchange (PLEX) have been frequently used with excellent results [4,16,19]. Noticeably, Xiao et al. (2021) reported no significant difference in clinical improvement between patients who received high-dose IVMP alone and those who received high-dose IVMP with IVIG or PLEX, suggesting the possibility of selecting treatment based on disease severity, economic circumstances, and availability. They also observed that patients with myelopathy were initially responsive to immunotherapy but had a higher rate of relapses during follow-up, highlighting the need for more aggressive and long-term medication for this specific category of patients. IVMP and IVIG have also been effective in patients with peripheral neuropathy [19,21]. Despite significant improvement with first-line immunotherapies in most studies, some patients reported severe disability, or even death [5,6]. One possible explanation is late diagnosis and delayed initiation of treatment [16].

Although most patients with autoimmune GFAP astrocytopathy present a monophasic course, relapses have been reported in 20–50%, 18%, and 12.1% of the cases according to previous studies [2,3,5,13,16,19,39]. In a study with pooled data from 324 patients, 41 out of 145 cases (28.3%) experienced clinical relapses [9]. Patients with relapses or with refractory disease activity are most commonly treated with a combination of oral steroids and a steroid-sparing, immunosuppressive agent, similar to MOG antibody disease and AQP4-NMOSD [40,41,42]. Steroids usually include prednisolone 60 mg/day (ranging between 20 and 60 mg/day, depending on weight) for 3 months, followed by a decrease of 10 mg/month until 10 mg, then 1 mg/month [13,16]. Faster tapering schemes have also been used [5,9]. In most patients, relapse may occur during the tapering phase of oral steroids or at discontinuation of prednisolone or steroid-sparing immunotherapy [2,9,19]. Relapses usually present as worsening of previously existing symptoms, onset of new symptoms, or both, though this is considered rare. Various and heterogeneous steroid-sparing immunotherapies have been used, sometimes along with oral steroids, including rituximab, mycophenolate mofetil, tacrolimus, azathioprine, and cyclophosphamide, all with promising immunosuppressant effects [2,9,13,39,43,44,45]. Flanagan et al. (2017) reported no relapses with mycophenolate, although two patients required increased doses [2]. Xiao et al. (2021) observed no relapses with tacrolimus and cyclophosphamide, while Zhang et al. (2023) reported a patient treated with tacrolimus who experienced frequent relapses, hence suggesting poor efficacy [9,39]. Rituximab, an anti-CD20 monoclonal antibody, has been successfully used for the treatment of steroid-resistant or relapsing GFAP astrocytopathy [43,44]. Although T-cell-mediated autoimmune syndromes generally do not respond as effectively to B-cell-depleting agents as antibody-mediated autoimmune syndromes, GFAP astrocytopathy seems to constitute an exception to this pattern. This highlights its complex pathophysiology, suggesting that several other mechanisms besides T-cell cytotoxicity are involved. Interestingly, a case of rituximab-resistant GFAP astrocytopathy treated successfully with Ofatumumab was described recently. Ofatumumab is another anti-CD20 monoclonal antibody that has been approved recently for the treatment of relapsing–remitting multiple sclerosis (RRMS) [45]. Accompanying NMDAR-IgG antibodies, AQP-4 antibodies, and neoplasms are considered negative prognostic factors and have been associated with poorer responses to first-line acute immunotherapies and higher rates of relapses [3,9]. Interestingly, Zhang et al. (2023) reported that viral infection identified in the serum is associated with a poorer short-term prognosis [39].

## 10. Conclusions

GFAP astrocytopathy is an immune-mediated disorder of the brain with occasional involvement of the spinal cord, mediated by CD8+ T-cells rather than by a direct pathogenic effect of anti-GFAP antibodies, yet its pathophysiology remains obscure. It is characterized by a broad clinical spectrum, though it most commonly manifests as meningoencephalitis with or without myelitis. Patients presenting with non-infectious encephalitis and meningeal signs should undergo CSF GFAP antibody tissue- and cell-based testing. Other characteristic clinical or paraclinical findings that should raise suspicion for GFAP astrocytopathy include subacute psychiatric or extrapyramidal syndromes, symptomatic or asymptomatic bilateral optic disk edema, brain linear perivascular radial contrast enhancement, and a favorable response to steroid treatment. The condition is often developed in the context of a malignancy or following an infection and its management includes high-dose intravenous corticosteroids, IVIG, and PLEX. Further studies are essential to deepen our understanding of the clinical, biological, and imaging characteristics of this recently described autoimmune disorder, as well as to shed light on its complex pathophysiology.

## Figures and Tables

**Table 1:** Clinical features of GFAP astrocytopathy.

**Clinical Features**	
Clinical Phenotype	(%)
Meningoencephalitis	23–55%
Meningoencephalomyelitis	21–40%
Encephalitis	13%
Encephalomyelitis	11%
Myelitis	5%
Meningitis	3%
Clinical Symptoms/Findings	
Fever	61–93%
Headache	55–79%
Decreased consciousness	38–79%
Meningeal signs	23–71%
Movement disturbances	40–64%
Psychiatric symptoms	23–36%
Autonomic dysfunction	20–57%
Visual symptoms	28–30%
Seizures	16–20%
Area postrema syndrome	11%
Respiratory failure	4–12%
Peripheral neuropathy	<5%

## Data Availability

Not applicable.
